# Intraoperative Ultrasound Guidance Is Associated with Clear Lumpectomy Margins for Breast Cancer: A Systematic Review and Meta-Analysis

**DOI:** 10.1371/journal.pone.0074028

**Published:** 2013-09-20

**Authors:** Hong Pan, Naping Wu, Hao Ding, Qiang Ding, Juncheng Dai, Lijun Ling, Lin Chen, Xiaoming Zha, Xiaoan Liu, Wenbin Zhou, Shui Wang

**Affiliations:** 1 Department of Breast Surgery, The First Affiliated Hospital with Nanjing Medical University, Nanjing, Jiangsu, China; 2 Department of Surgery, Baoying County Hospital, Yangzhou, Jiangsu, China; 3 Department of Epidemiology and Biostatistics, Nanjing Medical University School of Public Health, Nanjing, Jiangsu, China; Northwestern University Feinberg School of Medicine, United States of America

## Abstract

**Purpose:**

Margin status is one of the most important predictors of local recurrence after breast conserving surgery (BCS). Intraoperative ultrasound guidance (IOUS) has the potential to improve surgical accuracy for breast cancer. The purpose of the present meta-analysis was to determine the efficacy of IOUS in breast cancer surgery and to compare the margin status to that of the more traditional Guide wire localization (GWL) or palpation-guidance.

**Methods:**

We searched the database of PubMed for prospective and retrospective studies about the impact of IOUS on margin status of breast cancer, and a meta-analysis was conducted.

**Results:**

Of the 13 studies included, 8 were eligible for the impact of IOUS on margin status of non-palpable breast cancers, 4 were eligible for palpable breast cancers, and 1 was for both non-palpable and palpable breast cancers. The rate of negative margins of breast cancers in IOUS group was significantly higher than that in control group without IOUS (risk ratio (RR)  = 1.37, 95% confidence interval (CI)  = 1.18–1.59 from 7 prospective studies, odds ratio (OR)  = 2.75, 95% CI  = 1.66–4.55 from 4 retrospective studies). For non-palpable breast cancers, IOUS-guidance enabled a significantly higher rate of negative margins than that of GWL-guidance (RR  = 1.26, 95% CI  = 1.09–1.46 from 6 prospective studies; OR  = 1.45, 95% CI  = 0.86–2.43 from 2 retrospective studies). For palpable breast cancers, relative to control group without IOUS, the RR for IOUS associated negative margins was 2.36 (95% CI  = 1.26–4.43) from 2 prospective studies, the OR was 2.71 (95% CI  = 1.25–5.87) from 2 retrospective studies.

**Conclusion:**

This study strongly suggests that IOUS is an accurate method for localization of non-palpable and palpable breast cancers. It is an efficient method of obtaining high proportion of negative margins and optimum resection volumes in patients undergoing BCS.

## Introduction

Breast cancer is the most common malignancy among women in the world [1]. As the improvements in imaging techniques, the increased awareness of patients and widespread screening mammography, the number of women diagnosed with early stage breast cancer has increased during the past decades [2]. Breast conserving surgery (BCS) plus adjuvant radiotherapy has become the alternative treatment to mastectomy for early stage breast cancer because of equivalent survival [3], [4]. It is well known that obtaining negative surgical margins during BCS procedures is considered to be critically important in decreasing recurrence rates [5], [6].

The current focus is on improving the surgical accuracy of BCS, especially a higher rate of margin negative with smaller excision volume [7], [8]. Various techniques have been used to localize breast lesions during BCS. Guide wire localization (GWL) is a standard technique for localization of non-palpable breast lesions [9]. However, there are several disadvantages of GWL, including a miss rate up to 20% of cases, and the possibility of wire transaction, dislocation or migration. Besides, the insertion of the wire could be uncomfortable for patients [10], [11]. For palpable breast cancer, tumor excision is usually guided by preoperative diagnostic images and experience and tactile skills of the surgeons. However, palpation alone may be insufficient in differentiating between malignant tissues and surrounding tissues, especially in dense breasts. Palpation-guided surgery could lead high incidence of positive margins, ranging from 20% to 60% [12], [13].

Therefore, a procedure with a higher rate of negative margins and less discomfort for the patient would be preferable. Examining the breasts by Ultrasound (US) was first described by Wild and Neal [14]. Its application has expanded from preoperative assessment and diagnostic guidance to intraoperative localization of breast cancers. Intraoperative ultrasound guidance (IOUS) was developed in 1988 [15]. It is an ultrasound probe that is used to localize the breast tumor in the operating theatre. IOUS may improve surgical accuracy of breast cancer excision. Since its introduction, several studies were published on IOUS focusing on margin status for either non-palpable or non-palpable breast cancers.

Some studies suggested that IOUS could not increase the rate of complete tumor removal significantly compared with GWL [16], [17]. Some other studies demonstrated that IOUS-guided excision can significantly lower the proportion of positive margins of breast cancers than GWL-guided or palpable-guided excision for either non-palpable or palpable breast cancers. Besides, the technique is non-invasive, simple, safe and comfortable for patients [18], [19], [20].

To better evaluate whether IOUS is associated with clear lumpectomy margins for non-palpable and palpable breast cancers, we performed a systematic review and meta-analysis on IOUS focusing on margin status, compared with other traditional aiding techniques.

## Methods

### Search strategy

This systematic review and meta-analysis was performed as described previously [21], [22]. Relevant studies were selected by searching PubMed (update to March 31, 2013), using the following terms: breast cancer or breast neoplasm, and intraoperative ultrasound. The search was limited to those studies written in English. Three reviewers (Pan, Wu and Ding) independently evaluated titles and abstracts of identified articles. Potentially relevant articles were retrieved to review the full text. We also reviewed the references in the articles for possible inclusions.

In this study, we mainly focused on margin status of breast cancers. Margin status was divided into two categories: negative margin and positive margin. Negative margin was usually defined as having a microscopically tumor-free margin at least 1 mm, and a distance of less than 1 mm was considered to be positive margin in the identified articles. Studies that met the following criteria were included in the meta-analysis: (1) the diagnosis of breast cancer was confirmed histopathologically; (2) for meta-analysis of the association studies, patients who have received IOUS were considered as case patients, while patients who have not received IOUS were considered as control patients; (3) the impact of IOUS on margin status was evaluated; (4) sufficient data for estimating an odds ratio (OR) or risk ratio (RR) with 95% confidence interval (CI). Besides, only the one with the largest sample numbers was included for overlapping studies.

### Data extraction

Information was carefully extracted from all the eligible studies independently by three reviewers (Pan, Wu and Ding). Consensus was reached by discussion. The following variables were extracted from each study if available: first author's name, publication year, study design, types of breast cancer, the technique used to localize breast lesions (both case group and control group), number of cases and controls, and number of patients with negative margins.

### Statistical analysis

We conducted meta-analysis among women who diagnosed with either palpable breast cancer or non-palpable breast cancer jointly or separately. Crude ORs or RRs with 95% CI were used to assess the association between the different techniques used to localize breast lesions and the rate of negative margins. The between-study heterogeneity was tested with Q statistics [23]. The between-study heterogeneity was considered to be significantly only if *P*<0.10. When between-study heterogeneity was absent, the fixed-effects model (the Mantel-Haenszed method) [24] was used to calculate the pooled ORs. Otherwise, a random-effects model (the DerSimonian and Laird method) [25] was selected. The meta-analysis was performed as described previously [21], [22]. Publication bias was investigated by Funnel plots and Egger's linear regression, and *P*<0.05 was considered significant [26]. All analyses were performed using the software Stata version 11.0 (Stata Corporation, College Station, TX, USA).

## Results

### Description of the included studies

**Figure 1:**
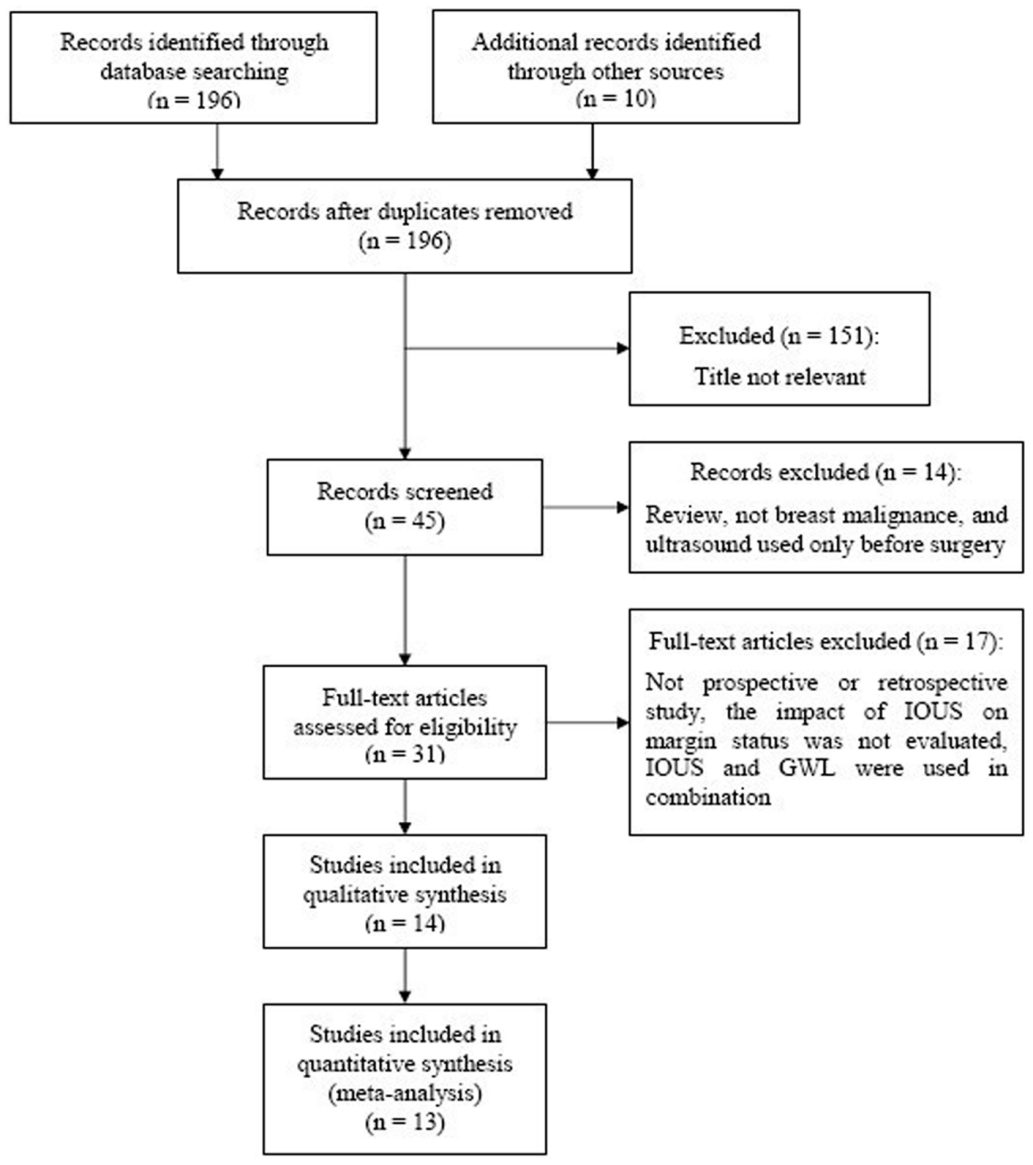
Selection of studies for the meta-analysis of IOUS associated negative margins of breast cancer.

Our initial search identified 196 potentially relevant studies, of which we screened the titles and abstracts. After full-text review of the 31 relevant studies, 13 studies [11], [16], [17], [18], [19], [20], [27], [28], [29], [30], [31], [32], [33] were included in this systematic review and meta-analysis (Fig. 1). The details of eligible studies about the efficacy of IOUS on margin status are shown in Table 1. Eight studies were prospective studies, and 5 were retrospective studies. All the eligible studies focused on the investigation of a possible improvement of margin status when IOUS was used during surgery, compared with surgery without IOUS. Of the 13 studies, 8 studies [11], [16], [17], [18], [19], [27], [28], [29] were carried out to investigate whether IOUS could enable a better margin clearance than GWL during non-palpable breast cancers excision. Four studies [20], [30], [31], [32] were eligible for the efficacy of IOUS on margin status of palpable breast cancer, compared with palpation alone. Besides, there is a study [33] evaluating the efficacy of IOUS regardless of whether the breast cancer is palpable or non-palpable.

**Table 1:** Characteristics of eligible studies about the efficacy of IOUS on margin status included in the study.

Author	Year	Design	Types of diseases	Presentation	Control	IOUS (n)		Control (n)	
						Margin (−)	Total	Margin (−)	Total
Rahusen FD	1999	Prospective	BC	Non-palpable	GWL	17	19	17	43
Snider HC	1999	Prospective	BC	Non-palpable	GWL	18	22	18	22
Rahusen FD	2002	Prospective	IBC	Non-palpable	GWL	24	27	12	22
Bennet IC	2005	Prospective	BC	Non-palpable	GWL	39	42	19	24
Haid A	2007	Prospective	BC	Non-palpable	GWL	242	299	38	61
James TA	2009	Retrospective	DCIS	Non-palpable	GWL	64	96	36	59
Krekel NMA	2011	Retrospective	IBC + DCIS component	Non-palpable	GWL	43	52	86	117
Barentsz MW	2012	Prospective	IBC	Non-palpable	GWL	112	120	129	138
Morre MM	2001	Prospective	IDBC	Palpable	Other	26	27	17	24
Davis KM	2011	Retrospective	IDBC	Palpable	Palpation-guide	20	22	26	44
Fisher CS	2011	Retrospective	IDBC	Palpable	Palpation-guide	66	73	104	124
Krekel NMA	2013	Prospective	IBC + DCIS component	Palpable	Palpation-guide	58	65	50	69
Eichler C	2012	Retrospective	BC	Both	Palpation-guide	81	84	137	166

BC, breast cancer; IBC, invasive breast cancer; IDBC, invasive ductal breast cancer; DCIS, ductal carcinoma in situ; IOUS, intraoperative ultrasound guidance; GWL, guide wire localization.

### Impact of IOUS on rates of negative margins of all breast cancers

For all prospective studies included, the rate of negative margins of breast cancers (including non-palpable and palpable breast cancers) in IOUS group (536/613) was significantly higher than that in control group without IOUS (300/403) (RR  = 1.63, 95% CI  = 1.10-2.42, *P* = 0.010 for heterogeneity) (Table 2).

**Table 2:** Summary ORs or RRs and 95% CI of IOUS associated negative margins.

Presentation	Types of study	Summary ORs/RRs	95% CI	*P* value for heterogeneity
Both	Prospective	1.63*	1.10–2.42	0.010
		1.37	1.18–1.59	0.117
	Retrospective	2.42*	1.45–3.19	0.122
		2.75	1.66–4.55	0.193
Non-palpable	Prospective	1.47*	0.98–2.22	0.030
		1.26	1.09–1.46	0.334
	Retrospective	1.45	0.86–2.43	0.583
Palpable	Prospective	2.36	1.26–4.43	0.361
	Retrospective	2.71	1.25–5.87	0.146

* ORs or RRs was meta-analyzed with publication bias.

**Figure 2:**
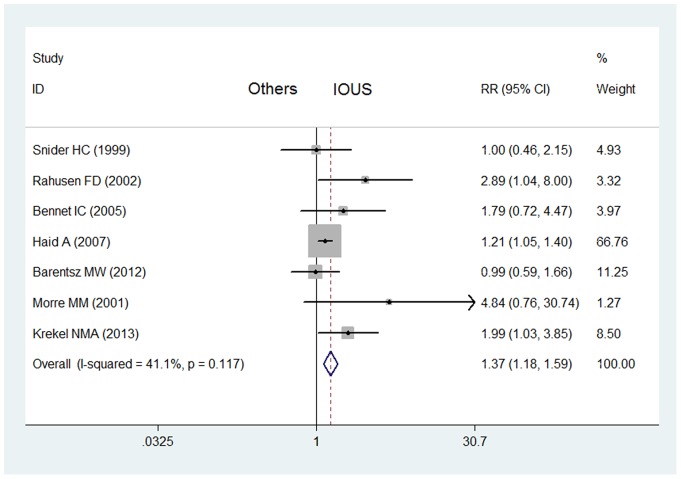
Impact of IOUS on rates of negative margins of all breast cancers (prospective studies). Forest plot for meta-analysis of prospective studies: rate of negative margins of all breast cancers (including non-palpable and palpable breast cancers) in IOUS group compared with that in control group without IOUS. The width of the horizontal line represents the 95% CI of the individual study, and the square proportional represents the weight of each study. The diamond represents the pooled RR and 95% CI.

The results of Funnel plots and Egger's linear regression showed that there was publication bias caused by the study [27] published in 1999 (*P*<0.05). After this study excluded, there is no publication bias (*P*>0.05). And the rate of negative margins of breast cancers in IOUS group (519/594) was still significantly higher than that in control group without IOUS (283/360) (RR  = 1.37, 95% CI  = 1.18–1.59, *P* = 0.117 for heterogeneity) (Fig. 2).

**Figure 3:**
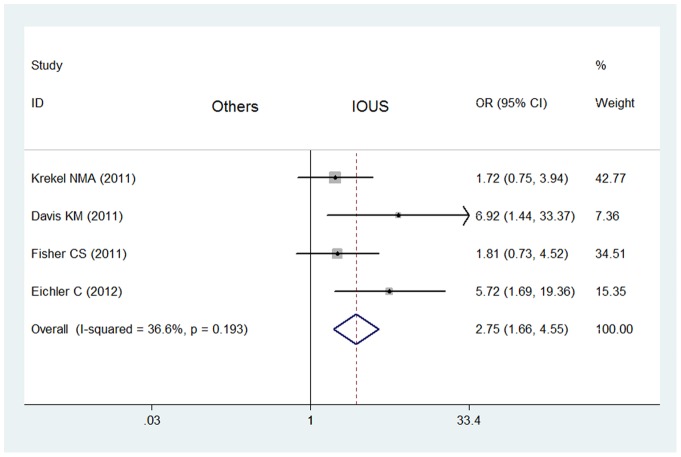
Impact of IOUS on rates of negative margins of all breast cancers (retrospective studies). Forest plot for meta-analysis of retrospective studies: rate of negative margins of breast cancers (including non-palpable and palpable breast cancers) in IOUS group compared with that in control group without IOUS. The width of the horizontal line represents the 95% CI of the individual study, and the square proportional represents the weight of each study. The diamond represents the pooled OR and 95% CI.

What's more, the meta-analysis of 5 retrospective studies included showed that the rate of negative margins of breast cancers in IOUS group (274/327) was significantly higher than that in control group without IOUS (389/510) (OR  = 2.42, 95% CI  = 1.45–3.19, *P* = 0.122 for heterogeneity) with publication bias (*P*<0.05) (Table 2). After the study was excluded [29], there is no publication bias (*P*>0.05). And the rate of negative margins of breast cancers in IOUS group (210/231) was still significantly higher than that in control group (353/451) without IOUS (OR  = 2.75, 95% CI  = 1.66–4.55, *P* = 0.193 for heterogeneity) without publication bias (Fig. 3).

### Impact of IOUS on rates of negative margins of non-palpable breast cancers

**Figure 4:**
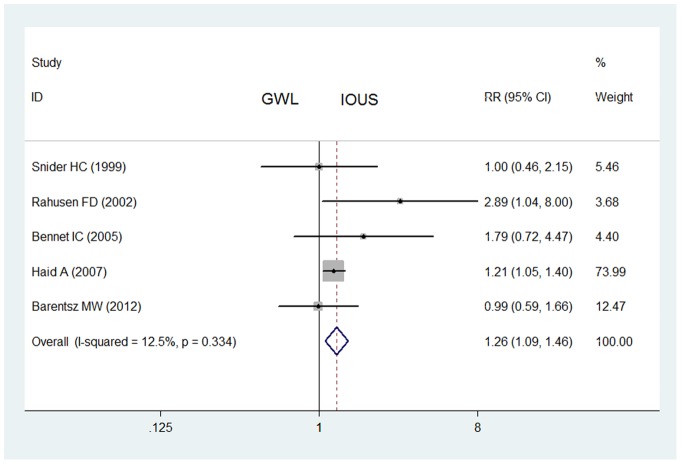
Impact of IOUS on rates of negative margins of non-palpable breast cancers (prospective studies). Forest plot for meta-analysis of prospective studies: rate of negative margins of non-palpable breast cancers in IOUS group compared with that in GWL group. The width of the horizontal line represents the 95% CI of the individual study, and the square proportional represents the weight of each study. The diamond represents the pooled RR and 95% CI.

For all prospective studies on non-palpable breast cancers, IOUS did not enable a significantly higher rate of negative margins (452/529) than that of GWL (233/310) (RR  = 1.47, 95% CI  = 0.98–2.22, *P* = 0.030 for heterogeneity) with publication bias (*P*<0.05) (Table 2). After one study was excluded [27], IOUS enabled a significantly higher rate of negative margins (435/510) than that of GWL (217/267) (RR  = 1.26, 95% CI  = 1.09–1.46, *P* = 0.334 for heterogeneity) without publication bias (Fig. 4).

**Figure 5:**
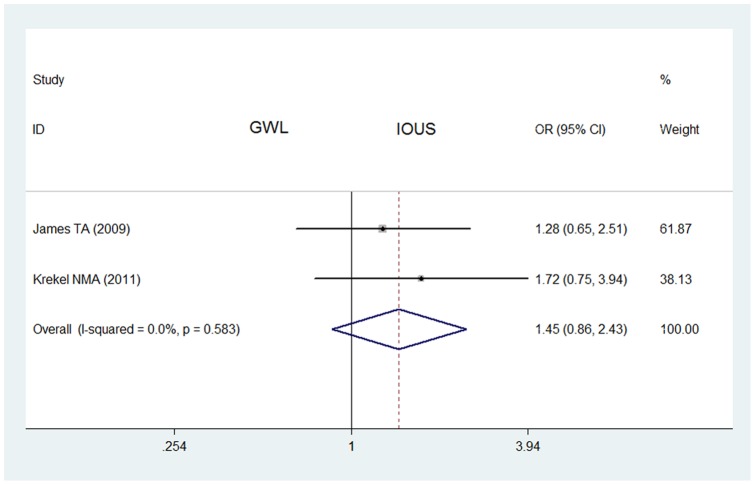
Impact of IOUS on rates of negative margins of non-palpable breast cancers (retrospective studies). Forest plot for meta-analysis of retrospective studies: rate of negative margins of non-palpable breast cancers in IOUS group compared with that in GWL group. The width of the horizontal line represents the 95% CI of the individual study, and the square proportional represents the weight of each study. The diamond represents the pooled OR and 95% CI.

The meta-analysis of 2 retrospective studies also showed that the rate of negative margins of non-palpable breast cancers in IOUS group (107/148) was not significantly higher than that in GWL group (122/176) (OR  = 1.45, 95% CI  = 0.86–2.43, *P* = 0.583 for heterogeneity) (Fig. 5).

### Impact of IOUS on rates of negative margins of palpable breast cancers

**Figure 6:**
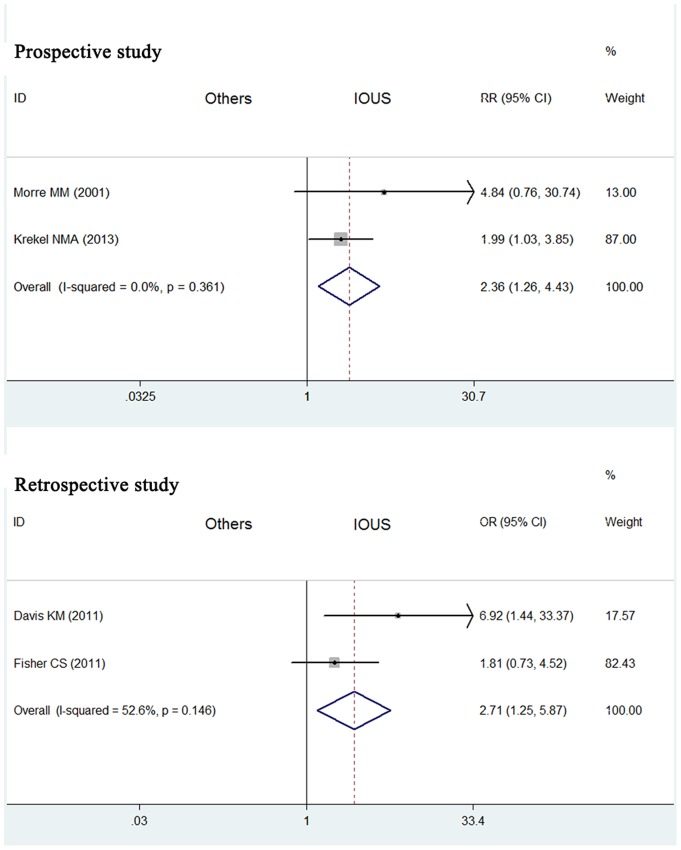
Impact of IOUS on rates of negative margins of palpable breast cancers. Forest plot for meta-analysis: rate of negative margins of palpable breast cancers in IOUS group compared with that in control group without IOUS. The width of the horizontal line represents the 95% CI of the individual study, and the square proportional represents the weight of each study. The diamond represents the pooled RR or OR and 95% CI.

Fig. 6 showed the results of meta-analysis of 2 prospective studies on the impact of IOUS on rates of negative margins of palpable breast cancers. Relative to control group without IOUS, the RR for IOUS associated negative margins was 2.36 (95% CI  = 1.26–4.43, *P* = 0.361 for heterogeneity) (Table 2). Besides, Fig. 6 also showed the results of meta-analysis of 2 retrospective studies. Relative to control group, the OR for IOUS associated negative margins was 2.71 (95% CI  = 1.25–5.87, *P* = 0.146 for heterogeneity).

## Discussion

BCS is a landmark for treating patients with early stage breast cancer. Up to now, different management procedures have been applied to obtain negative margins for patients who have undergone BCS [34]. There is still no preoperative assessment that can ensure the clear lumpectomy margins at the initial operation. GWL is the standard technique for tumor localization of non-palpable breast cancers [9]. And excision of a palpable breast cancer is usually guided by the intraoperative tactile skills of the surgeon. IOUS enables the surgeon to optimally position the incision on the breast and to operate under direct vision. It is proposed to replace GWL, with evidence from our present meta-analysis.

Our present systematic review and meta-analysis sums up the previous studies on the impact of IOUS on margins status of breast cancer. The results demonstrated a statistically significant increase in the incidence of pathologically negative margins with the use of IOUS, for both non-palpable and palpable breast cancers.

Apart from this, cosmetic outcome must be taken into account. The goal of attaining a negative margin must be balanced with cosmetic outcome. Removal of a larger volume of tissue is not advocated because of a poorer cosmetic outcome [35]. Because of the inconsistency of tumor size, the excess breast tissue resection was better determined using the calculated resection ratio (CRR), representing a comparison of the total resection volume to the optimal resection volume [16]. It is notable that IOUS-guided surgery resulted in a reduced CRR compared with surgery without IOUS, thereby improving cosmetic outcome and increasing patients' satisfaction and quality of life [16], [20]. Unfortunately, CRR can't be meta-analyzed in this study, due to rarely few studies having provided the data.

In addition, the average cost of an IOUS was much less than GWL [29]. The cost differential between the procedures would favor IOUS as a means of localization when feasible. On the other hand, IOUS can be performed by breast surgeons not depending on radiology. It also avoids the need for a separate invasive procedure.

However, it can't be ignored that the use of IOUS to localization of breast cancer must be tailored to each individual case, as there remain situations where GWL will be more appropriate [36]. Many mammographically detected lesions, such as microcalcifications, are not visible by ultrasound. So IOUS alone is not a feasible alterative for some special patients. Thus, it may be necessary to combine IOUS with GWL in certain cases. The results of a previous study demonstrated that IOUS can facilitate the surgeon in performing GWL-guided breast procedures for mammographic abnormalities. The combined technique may increase the rate of negative margins and improve cosmoses [36].

In addition to IOUS, other alternative methods of intraoperative localization for breast lesions have been developed recently. Radioguided occult lesion localization (ROLL) has been used, obtaining good results with respect to GWL [37], [38]. Radioactive-labeled tracer was injected around the target lesion and a gamma probe was used to guide the excision in the operating room, similar to radio-guided sentinel lymph node biopsy [39]. However, during ROLL procedure, tracer injection involves difficulties in precision, in determining the amount to be injected, and in preventing spreading to the adjacent breast tissue.

There are limitations to this systematic review and meta-analysis that must be acknowledged. First, of the 13 studies, breast cancer types of patients participating in different trials were inconsistent. And the sample size of some studies is rarely small. Second, relevant baseline information regarding preoperative imaging measurements was not available in the studies included. Third, the present results were based on unadjusted ORs and RRs, and more precise estimation may be adjusted by other potential covariates, such as age, race, menopausal status and so on.

## Conclusion

This systematic review and meta-analysis strongly suggests that IOUS is an accurate method for localization of non-palpable and palpable breast cancer. It is an efficient method of obtaining higher proportion of negative margins and optimum resection volumes in patients undergoing BCS. It may be useful to use IOUS and GWL in combination in some situation.

## Supporting Information

Checklist S1 **PRISMA Checklist.**
(DOC)
